# Warfarin Dosing Requirement According to Body Mass Index

**DOI:** 10.7759/cureus.11047

**Published:** 2020-10-19

**Authors:** Abdullah Alshammari, Abdullah Altuwayjiri, Ziad Alshaharani, Rami Bustami, Hind S Almodaimegh

**Affiliations:** 1 Pharmaceutical Care Department, King Abdullah Medical City, Makkah, SAU; 2 Pharmaceutical Department, King Fahad Medical City, Riyadh, SAU; 3 College of Pharmacy, King Saud Bin Abdulaziz University for Health Science, Riyadh, SAU; 4 College of Pharmacy, Alfaisal University, Riyadh, SAU; 5 Pharmaceutical Care Department, Ministry of National Guard Health Affaire - King Abdulaziz Medical City, Riyadh, SAU

**Keywords:** warfarin, vitamin k antagonist, anticoagulant therapy, obesity, weight

## Abstract

Introduction

Many factors affect the required dose of warfarin, including body weight, which is mentioned only in a few studies. Our study was conducted using body mass index (BMI) to assess the requirements for warfarin dosing.

Methods

A retrospective study was conducted that included adults who used warfarin for more than three months, with at least two consecutive international ratio (INR) readings within the therapeutic range.

Results

Over 301 patients were included; the 20% higher dose of warfarin was required in obese patients than normal BMI and overweight patients (32.2 ± 15.2 vs. 27.4 ±17.3 and 26.8 ± 12.7; p=0.013, respectively).

Conclusion

Obese patients required a higher dose than other patients, which should be considered when initiating or adjusting the warfarin dose.

## Introduction

Warfarin exerts its activity by inhibiting vitamin K-dependent blood clotting proteins that affect the coagulation cascade factors II, VII, IX, and X. This makes warfarin a very effective antithrombotic agent to prevent and treat thromboembolic complications [1-4].

The international normalized ratio (INR) measures the efficacy and safety of warfarin. Achieving the therapeutic range is affected by many inter- and intra-patient characteristics. Several factors affect warfarin response, including medication interactions, diet, and acute illness. Age, gender, genetic polymorphism, and obesity can affect a patient's required dose of warfarin [3, 5-7].

The incidence of obesity is increasing around the world. In the USA, approximately 36.5% of the population is obese, while in Saudi Arabia, the prevalence of obesity is about 28.7%. Obesity is considered to be a risk factor for developing thrombosis, especially deep vein thrombosis (DVT) and pulmonary embolism (PE). It also increases the risk of atrial fibrillation [3, 8-11].

The lack of a standardized warfarin dosing regimen, and variation in patients' characteristics, makes defining the requirement of warfarin dose difficult. Therefore, in this study, we aimed to assess the effect of body weight, and other patient characteristics, on the average weekly dose of warfarin required to stabilize therapeutic INR score over a period of at least three months.

## Materials and methods

A retrospective cohort study including adult patients (age ≥ 18 years) admitted to King Abdulaziz Medical City, Riyadh, Saudi Arabia, between January 2014 and June 2017. By reviewing patient charts, patients were selected who had been on warfarin for at least three months, who had at least two therapeutic INR scores within the normal range over months, and who received a stable dose of warfarin. Demographic data and information about various clinical factors were collected, including age, gender, height, weight, body mass index (BMI), warfarin dose, and INR.

Patients included in the study were adults aged 18 years and over who had been on warfarin for at least three months with a therapeutic INR level. Patients with conditions other than DVT, PE, and atrial fibrillation were excluded.

Descriptive statistical analyses were performed on the study sample. Continuous variables were summarized using means ± SD, medians, and interquartile ranges (IQR). Proportions were used for categorical variables. Average warfarin dose was evaluated and compared against several demographic and clinical factors, including age, BMI, and gender, using Student’s t-test/the Mann-Whitney U test, or one-way ANOVA/the Kruskal-Wallis test. A multivariate linear regression model was used to identify factors associated with warfarin dosing. Statistical significance was considered at p<0.05. All statistical analyses were performed using SPSS 21.0 (IBM Inc., Armonk, USA).

## Results

A total of 301 subjects were included in the study. Descriptive statistics are given in Table 1. The respondents' age distribution was as follows: 15% of participants in the age group 18-44, 33.9% in the age group 45-65, and 51.2% in the age group above 65. Most patients were female (56.5%). The vast majority of patients were overweight or obese (85%). Twenty-six percent of patients had DVT, 18% had PE, and 67% had atrial fibrillation. Mean ± SD INR was 2.4 ± 0.3.

**Table 1:** Patients' characteristics (n=301)

Factor	Value (%)
Gender n (%)	
Female	170 (56.5)
Male	131 (43.5)
Age mean ± SD	61.7 ± 15.1
Median (interquartile range)	66 (53–74)
Age group (years) n (%)	
18–44	45 (15.0)
45–65	102 (33.9)
Above 65	154 (51.2)
Weight mean ± SD	84.1 ± 39.9
Median (interquartile range)	80 (68.9–92.8)
Height mean ± SD	160.3 ± 9.8
Median (interquartile range)	160 (153–167)
BMI category, n (%)	
Normal	46 (15.3)
Overweight	79 (26.2)
Obese	176 (58.5)
Deep vein thrombosis, n (%)	77 (25.6)
Pulmonary embolism, n (%)	53 (17.6)
Atrial fibrillation, n (%)	203 (67.4)
INR mean ± SD	2.4 ± 0.3
Median (interquartile range)	2.4 (2.2–2.6)

The results show in Table 2 that the weekly dose of warfarin was significantly lower in older patients (above 65 years), 25.4 ± 11.5 versus 39.7 ± 18.6, and 32.8 ± 15.7 for those aged 18-44 and 45-65 years, respectively (p<0.001). Obese patients had a significantly higher dose of warfarin than those with a normal or overweight BMI: 32.2 ± 15.2 versus 27.4 ± 17.3 (normal) and 26.8 ± 12.7 (overweight) (p=0.013). 

**Table 2:** Average weekly dose of warfarin by patients' characteristics

	N	Mean ± SD	Median (interquartile range)	p
All patients	301	30.0 ± 15.2	28.0 (20.0–37.5)	
Age group				<0.001
18–44	45	39.7 ± 18.6	35.0 (26.0–50.8)	
45–65	102	32.8 ± 15.7	31.5 (21.0–39.0)	
Above 65	154	25.4 ± 11.5	23.0 (17.5–35.0)	
BMI group				0.013
Normal	46	27.4 ± 17.3	22.3 (16.5–35.0)	
Overweight	79	26.8 ± 12.7	24.5 (17.5–35.0)	
Obese	176	32.2 ± 15.2	28.0 (21.0–41.5)	
Gender				0.43
Female	170	29.4 ± 15.2	28.0 (20.5–37.5)	
Male	131	30.8 ± 15.0	28.0 (20.0–38.5)	

## Discussion

The study aimed to assess the weekly required dose of warfarin for patients in three different BMI categories. Most patients were obese (BMI>30 kg/m2). Comparing obese patients with those in the other two groups (normal and overweight), obese patients required a statistically significantly higher warfarin dose by around 20%. 

A retrospective study by Mueller et al. assessed the total weekly dose of warfarin required by 831 patients who were stable and received the same dose for at least 30 days. Results showed that the weekly dose of warfarin required by morbidly obese patients was statistically significantly higher than that of normal body weight by around 20% [5].

In addition to other factors, high body weight is associated with an increasing requirement for warfarin, and this should be considered when initiating and adjusting the dose. Hospitalized patients who used warfarin for the first time for more than four consecutive days were categorized by body weight, and their dose of warfarin was managed by pharmacy staff. Out of 211 patients included in a retrospective study, the percentage who achieved a therapeutic INR was significantly different between the groups: 71.1% of normal body weight achieved the goal than 42.3% of obese patients and 38% of morbidly obese patients. Moreover, morbidly obese and obese patients required a higher average daily dose of warfarin compared to patients with normal weight (7.6 ± 0.5 mg, 6.6 ± 0.3 mg, and 5 ± 0.3 mg, respectively). The time taken for patients to achieve a therapeutic INR level was also longer for morbidly obese and obese patients than for normal-weight patients (10 days morbidly for obese, eight days for obese, and six days for normal) [3].

It is well known that warfarin interacts with dietary factors, including vitamin K. Therefore, when starting warfarin treatment and adjusting warfarin dose, the patient’s diet should be considered. One prospective study of 146 patients aimed to identify patient-specific factors that contributed to the required warfarin dose. An advantage of this study is that it used a structured questionnaire to estimate patients’ vitamin K intake. Patients included in the study had maintained a therapeutic INR level for at least four weeks and received a stable warfarin dose. Weight, especially weighing more than 90 kg, was one of five factors that contributed to an increasing required warfarin dose [4].

A study by Yoo et al. included 321 stroke patients who had achieved therapeutic INR levels at least three times in a row. The study results demonstrated that increasing body weight increases the warfarin dose required to achieve a therapeutic INR level. Based on these results, a protocol was developed for prescribing an appropriate initial dose of warfarin. This protocol recommends that patients aged 80 years and older, or who have a body weight less than 55 kg, should receive a lower initial maintenance dose of warfarin (3 mg); patients with a body weight of more than 55 kg, or who are younger than 55 years should receive a higher initial maintenance dose of warfarin (10 mg), and any patient between these ranges should receive a standard initial maintenance dose of warfarin of between 3 and 7 mg [12-13]. 

Most of the previous studies assessed the effect of warfarin in acute settings as initial therapy or for chronic settings where warfarin was used for a period of less than one month. Our study focused on assessing the required weekly dose of warfarin in patients who are stable on warfarin for at least three months. Additionally, previous studies showed the effect of weight on increasing the warfarin requirement. The proposed mechanism for these results is related to the volume of distribution, and clearance is positively correlated with coagulation factor levels. Therefore, in obese patients, the increase in weight would lead to an increase in the volume of distribution and clearance of warfarin, with that the levels of coagulation factors would increase; hence more doses of warfarin would be required [14].

Even though we included only those patients with a maintained therapeutic INR level, and who received a stable dose of warfarin over three months, there are several limitations of the study. For example, the study was retrospective and took place in only one center. There are also several variables that could not be assessed, such as drug-drug interactions, diet, and genetic polymorphism. 

## Conclusions

The development of a protocol for warfarin dosing should take all variables that may affect warfarin response into account so that future patients aiming to achieve therapeutic INR levels do not suffer from over-anticoagulation or sub-anticoagulation. One of these variables is body weight since we have shown that obese patients require higher warfarin doses to achieve a therapeutic INR level.
